# A Longer T_peak_-T_end_ Interval Is Associated with a Higher Risk of Death: A Meta-Analysis

**DOI:** 10.3390/jcm12030992

**Published:** 2023-01-28

**Authors:** Cathrin Caroline Braun, Matthias Daniel Zink, Sophie Gozdowsky, Julie Martha Hoffmann, Nadine Hochhausen, Anna Bettina Röhl, Stefan Kurt Beckers, Felix Kork

**Affiliations:** 1 Department of Anesthesiology, Medical Faculty, RWTH Aachen University, Pauwelsstraße 30, 52074 Aachen, Germany; 2 Department of Cardiology, Medical Faculty, RWTH Aachen University, Pauwelsstraße 30, 52074 Aachen, Germany; 3 Medical Management, Emergency Medical Service, Berlin Fire Brigade, 10150 Berlin, Germany; 4 Aachen Institute of Emergency Medicine and Civil Security, Medical Faculty, RWTH Aachen University, Pauwelsstraße 30, 52074 Aachen, Germany

**Keywords:** T_peak_-T_end_ interval, dispersion of repolarization, cardiovascular disease, all-cause mortality, risk stratification

## Abstract

A noninvasive tool for cardiovascular risk stratification has not yet been established in the clinical routine analysis. Previous studies suggest a prolonged T_peak_-T_end_ interval (the interval from the peak to the end of the T-wave) to be predictive of death. This meta-analysis was designed to systematically evaluate the association of the T_peak_-T_end_ interval with mortality outcomes. Medline (via PubMed), Embase and the Cochrane Library were searched from 1 January 2008 to 21 July 2020 for articles reporting the ascertainment of the T_peak_-T_end_ interval and observation of all-cause-mortality. The search yielded 1920 citations, of which 133 full-texts were retrieved and 29 observational studies involving 23,114 patients met the final criteria. All-cause deaths had longer T_peak_-T_end_ intervals compared to survivors by a standardized mean difference of 0.41 (95% CI 0.23–0.58) and patients with a long T_peak_-T_end_ interval had a higher risk of all-cause death compared to patients with a short T_peak_-T_end_ interval by an overall odds ratio of 2.33 (95% CI 1.57–3.45). Heart rate correction, electrocardiographic (ECG) measurement methods and the selection of ECG leads were major sources of heterogeneity. Subgroup analyses revealed that heart rate correction did not affect the association of the T_peak_-T_end_ interval with mortality outcomes, whereas this finding was not evident in all measurement methods. The T_peak_-T_end_ interval was found to be significantly associated with all-cause mortality. Further studies are warranted to confirm the prognostic value of the T_peak_-T_end_ interval.

## 1. Introduction

Cardiovascular diseases (CVDs) affect the entire arterial circulation, while coronary heart disease (CHD) is the leading cause of death [1]. The prevalence of CHD is considered 6.7% in adults aged ≥ 20 years in the United States and around 35% of patients die as a result of a coronary event in a given year [2]. Despite an overall decline in CHD and CVD mortality rates over the last decades, this trend does not apply to all ethnicities, socioeconomic groups or geographic regions [2,3,4]. Adaptions in medical treatments and risk factors noticeably reduced deaths, however, risk stratification remains difficult [5].

Traditional electrocardiographic (ECG) findings of abnormal ventricular repolarization have been established as prognostic risk markers for cardiac events, i.e., arrhythmias as well as cardiac and all-cause death [6,7,8]. Whereas the QT interval reflects the total duration of ventricular depolarization and repolarization, the interval from the peak to the end of the T wave (T_peak_-T_end_ interval) supposedly correlates with the dispersion of the repolarization process [9]. 

Previous studies suggest a prolonged T_peak_-T_end_ interval to be predictive of death. Tse et al. (2017) already performed meta-analyses of these investigations by pooling together the odds or hazard ratios regarding arrhythmic and mortality outcomes [10]. However, several substantial shortcomings were debated controversially, such as an insufficient consideration of differing ECG measurement approaches or the heart rate correction of the Tpeak-Tend interval [11,12]. Additionally, the inclusion of six studies examining the risk in patients with Brugada syndrome is likely to have biased the results based on congenital repolarization disorders. Compared to the meta-analysis of Tse et al. we excluded patients with Brugada syndrome and focused primarily on the predictive value of prolonged Tpeak-Tend intervals for all-cause death. In addition, several subsequent studies have not been taken into consideration so far. Individual studies identified a prolonged Tpeak-Tend interval to be a surrogate marker for diffuse myocardial injury and myocardial damage without ST-elevation (NSTEMI) [13,14], which may potentially lead to arrhythmias and death [15,16,17]. Therefore, we conducted a meta-analysis investigating the association of the T_peak_-T_end_ interval with all-cause mortality, troponin elevation and the incidence of NSTEMI.

## 2. Materials and Methods

This meta-analysis was performed in accordance with current Cochrane standards [18] and reported based on the Preferred Reporting Items for Systematic Reviews and Meta-Analyses (PRISMA) statement [19,20]. Methodological details of the analysis were specified in advance and the resulting study protocol was registered at PROSPERO (CRD42020131622).

### 2.1. Eligibility Criteria and Search Strategy

Before the search was initiated, a precise clinical research question was developed using the population, intervention, comparisons and outcomes (PICO) format [21]. Potential studies were required to address the following study-specific elements of interest: Individuals of any age receiving an ECG recording with ascertainment of the T_peak_-T_end_ interval and observation of all-cause mortality, troponin elevation or NSTEMI as outcome. 

We performed a systematic literature search of studies published between 1 January 2008 and 21 July 2020 in MEDLINE (via PubMed), Embase and the Cochrane Library. As standard of care rapidly improves over time (specifically for this analysis with regards to precision of measurement and patients’ outcome), we restricted the literature search to twelve years into the past from the time it was conducted. The search terms used to identify relevant studies were the following: “Tpeak-Tend” OR “Tpeak-Tend” OR “Tp-Te” OR “Tp-Te” OR “Tpeak-end” OR “Tp-e” OR “T(peak)-T(end)” OR “T wave peak-to-end” OR “T peak-T end“ OR “TPEc“ OR “T-peak to T-end“ OR “Tpeak-to-Tend“ OR “T-Wave Peak to T-Wave End” OR “TpTe”. The search for keywords was limited to titles and abstracts, but no limits were applied for language. In the online supplement, the full electronic search strategy for MEDLINE via PubMed is presented (Resource S1). A manually screening of the reference lists of articles to identify additional matching studies complemented the electronic search. 

### 2.2. Study Selection Process

The findings were imported into a reference managing software (Endnote for Windows, version X9.3.3, Clarivate Analytics, Philadelphia, PA, USA) and duplicates were removed. All reports of retrospective and prospective observational studies (cohort studies, case control studies and cross sectional studies) in humans were included if the T_peak_-T_end_ interval duration was determined and either all-cause mortality, troponin elevation or NSTEMI were reported as endpoint and odds ratio (OR) or hazard ratio (HR) including the 95% confidence intervals (CIs), ROC curves or raw data necessary to calculate these were described. Both conference abstracts and journal articles were eligible for inclusion. Potential sample overlap was handled as follows: (1) if there was a corresponding article to a conference abstract that likely included the same or parts of the same sample population reported in the conference abstract (based on the time of publication and reported recruitment period), both the conference abstract and the journal article are reported but only the data from the journal article was introduced in the analysis; (2) if there was only a conference abstract without a corresponding journal article, we included the data of the conference abstract. Studies published in other languages than English that could not be translated by us or known associates, as well as review articles, letters and comments were excluded. 

Screening of abstracts was performed independently by two members of the study team (CCB, SG; interrater agreement Cohen’s kappa 0.72) and relevant full-texts were then assessed for eligibility twice. Any disagreement between the reviewers was solved by full-text retrieval of the respective study to avoid that any potentially eligible investigation was skipped in the full-text review.

### 2.3. Data Extraction and Management

Data from each study were extracted using a prespecified and piloted spreadsheet in Microsoft Excel. Each article was given an internal record identifier and the spreadsheet was structured as followed: the first section contained general information about the article including type of work (journal article or conference abstract) and the study design of the study. This section was followed by publication details including the first author’s surname, the year of publication as well as the country the study was conducted in. Going further into detail, in the next section we documented extracted data on the population that was investigated in the article, including sample size, number of patients and controls, sex distribution of the population, age distribution of the population and whether the investigated population suffered from a certain condition that characterizes this population (e.g., transcatheter aortic valve replacement patients). The following section contained characteristics regarding the ECG measurement conducted in the study, how was the T_peak_-T_end_ interval defined, what method was used to measure the T_peak_-T_end_ interval, in which leads was this measured, and whether reported interval lengths were heart rate corrected. After these basic study characteristics, the data extraction sheet continued with three respective sections for each investigated outcome endpoint: All-cause death, NSTEMI, and troponin change. For all-cause death, we extracted time of follow-up reported, number of survivors and deaths with respective mean T_peak_-T_end_ intervals, number of survivors and deaths with short or long T_peak_-T_end_ interval including the T_peak_-T_end_ cutoff lengths, reported odds and hazard ratios, whether these were derived from univariate or multivariate analyses, whether an receiver operating characteristic analysis was conducted with respective cutoff, sensitivity and specificity, positive predictive value and negative predictive value. Sections for endpoints NSTEMI and troponin change were similar.

Data extraction was conducted by one reviewer (CCB) and checked afterwards by a second reviewer (FK). Discrepancies were resolved by discussion between both reviewers. If one of the reviewer suspected data needed for meta-analysis to be available although not reported in the publication, corresponding authors of potentially eligible and included studies were contacted via e-mail and asked whether they could provide the unreported data (summary or individual de-identified data) for the purpose to be included in this meta-analysis. For each request, a minimum response time of 30 days was granted. 

### 2.4. Risk of Bias Assessment 

We evaluated the quality of primary studies applying an adapted version of the Newcastle-Ottawa Quality Assessment Scale for observational studies [22]. A quality score, ranging from 0–9 stars for cohort studies and 0–8 stars for case-control studies and cross-sectional studies, was calculated based on three major domains: selection of study groups, comparability of study groups and assessment of outcome or exposure. Age and sex of the study participants were defined as the most important covariates regarding comparability. An adapted form of the scale by Herzog et al. was used to provide quality assessment for one cross-sectional study [23]. The evaluation was completed by one reviewer (CCB) and was then verified by another team member (FK). Quality scores indicating poorer methodological quality were not considered as an exclusion criterion.

### 2.5. Assessment of Heterogeneity and Data Synthesis 

All analyses were conducted using the meta-package [24] for R 3.5.2 (www.r-project.org, accessed on 11 December 2022) for MacOS. Heterogeneity was assessed using the I^2^ statistic from chi-squared test. Due to a priori assumption of high clinical heterogeneity across trials due to different study populations, ECG leads used in measurements and types of death subsumed in all-cause mortality, we always used the appropriate random effects models. For comparing T_peak_-T_end_ intervals (milliseconds) in all-cause deaths and survivors, a standardized mean difference estimate was used to adjust for different types of T_peak_-T_end_ measurement methods (tangent method, tail method, Marquette 12SL algorithm, other computer-based measurements). To compare all-cause death in patients with longer versus shorter T_peak_-T_end_ intervals, unadjusted odds ratios using the DerSimonian-Laird method were applied. For studies reporting areas under the receiver operating characteristic (AUROC) curve with sensitivity, specificity and corresponding cutoff, the confusion matrix was back-calculated and a summary ROC curve was estimated using the “diagmeta” package for R. Subgroup analyses and sensitivity analyses were conducted post-hoc induced by the reported data of the original studies. Subgrouping was performed for groups that may present differences in the associations of the T_peak_-T_end_ interval with the outcome all-cause death: heart rate correction vs. no heart rate correction and different technical measurement methods of the T_peak_-T_end_ interval. Sensitivity analyses were conducted to account for possible bias by study design (prospective studies only) and study quality (Newcastle-Ottawa Quality Assessment Scale score greater 6).

## 3. Results

### 3.1. Description of Studies

**Figure 1:**
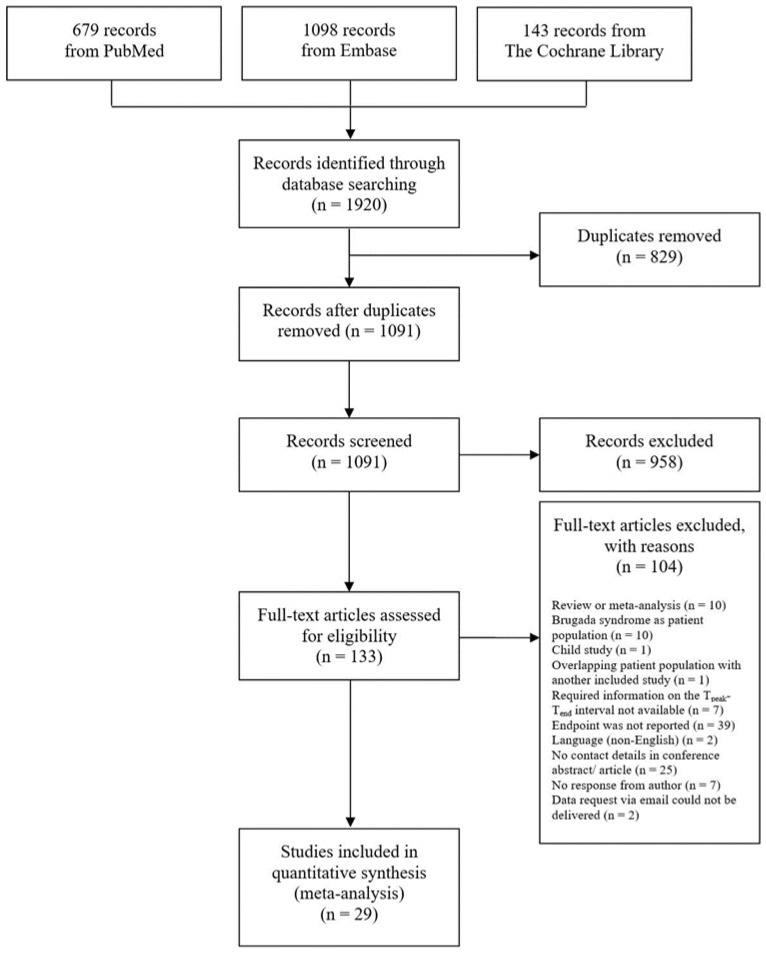
Flow chart of the search strategy and study selection process.

The search and selection process of studies is presented in Figure 1. 

The searching of Medline, Embase and the Cochrane Library provided a total of 1920 records. After deleting 829 duplicates, the title and abstract of a total of 1091 records were screened for eligibility. Of these, 958 records were non-compliant with the inclusion criteria and were therefore discarded and the remaining 133 records underwent full-text review. No additional studies could be identified via the examination of the reference lists. One study was excluded from the analysis, because the study population overlapped to a large extent with another study included in the analysis. Finally, 29 studies involving a total of 23,114 patients met the inclusion criteria. Table 1 provides an overview of the characteristics of the included studies; the quality assessment of the studies is presented in Table S1. Of the 29 studies included, 29 reported the primary endpoint all-cause mortality and none reported the secondary endpoints increase in troponin or NSTEMI. 

All included studies were observational studies published in English, originating mainly from Turkey (*n* = 6), the USA (*n* = 6), and Italy (*n* = 5). Among them were prospective (*n* = 15) and retrospective (*n* = 8) cohort studies, as well as case-control studies (*n* = 5) and one cross-sectional study (*n* = 1). The publication years ranged from 2009 to 2020, the majority was published in 2018 (*n* = 6). The most frequently examined populations were patients with cardiomyopathy/heart failure (*n* = 8), ST-segment elevation myocardial infarction (STEMI) (*n* = 6), cardiovascular diseases (*n* = 6) and other diseases (*n* = 7). Furthermore, two studies assessed the T_peak_-T_end_ interval in the general population. The ascertainment of the T_peak_-T_end_ interval presented as follows: 11 studies used precordial leads and again nine studies of these focused on leads V4-V6. Three studies assessed a combination of limb and precordial leads, two studies assessed leads with limited ST-segment deviation and five studies measured the T_peak_-T_end_ interval in all 12 leads. Additionally, five studies reported to have chosen the lead with the longest T_peak_-T_end_ interval. Studies determined the T_peak_-T_end_ interval applying four different methods: The tangent method (*n* = 6), the tail method (*n* = 4), an automatic measurement with the GE Healthcare Marquette 12SL algorithm (*n* = 5) and an automatic measurement using different software (*n* = 3). Furthermore, one study compared the tangent and tail method. Six studies did not report the leads for measuring the T_peak_-T_end_ interval and 11 studies did not report the method of measurement.

### 3.2. Association of T_peak_-T_end_ Interval with Mortality

#### The T_peak_-T_end_ Interval in Comparison between Survivors and Non-Survivors 

**Figure 2:**
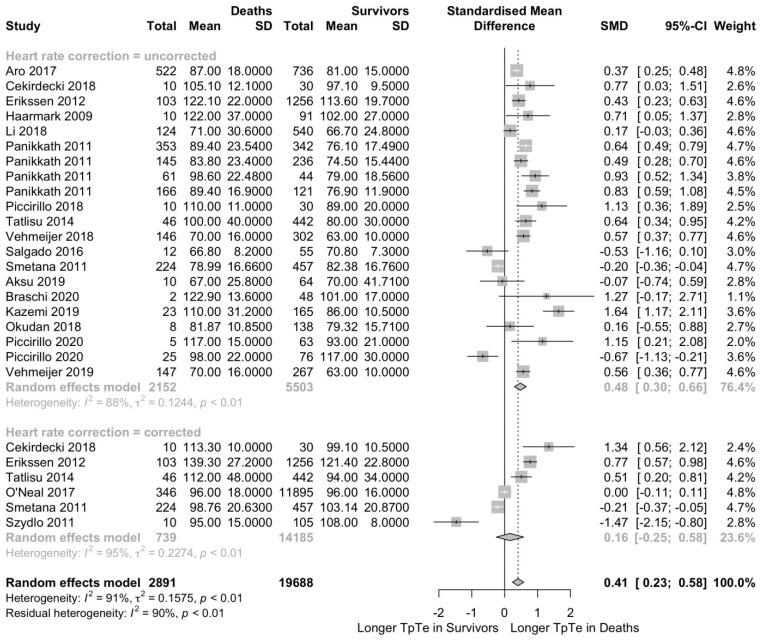
T_peak_-T_end_ interval is longer in patients who died compared to survivors. The forest plot shows the data from 20,011 patients from 21 studies, comparing the T_peak_-T_end_ interval in deaths and survivors. Patients who died had a longer T_peak_-T_end_ interval by a standard mean difference (SMD) of 0.41. T_p_T_e_ = T_peak_-T_end_ interval; SD [25,27,29,30,31,32,34,35,37,39,40,41,42,43,46,49,51,52,53].

The T_peak_-T_end_ interval was available for 27 patient populations in 16 articles and five conference abstracts, reporting data for 20,011 patients with 2508 all-cause deaths. We contacted eight investigators for additional data on the T_peak_-T_end_ interval, but only two authors provided the requested data. Cardiovascular diseases and STEMI represented the most frequent patient populations in this analysis with seven and six populations, respectively. Overall effect sizes for mortality outcomes are presented in Figure 2. The estimated overall standardized mean difference was 0.41 (95% CI 0.23–0.58; *p* < 0.0001; I^2^ = 91%; *p* < 0.01), indicating a statistically significant longer T_peak_-T_end_ interval in all-cause deaths with a moderate effect size.

A subgroup analysis was performed comparing 19 studies reporting an uncorrected T_peak_-T_end_ interval in 7655 patients versus six studies reporting a heart rate-corrected T_peak_-T_end_ interval in 14,924 patients. In both subgroups, all-cause deaths had a longer T_peak_-T_end_ interval: All-cause deaths had a longer uncorrected T_peak_-T_end_ interval by a standardized mean difference of 0.48 (95% CI 0.30–0.66; *p* < 0.0001; I^2^ = 88%; *p* < 0.01; Figure 2) and in tendency longer heart rate corrected T_peak_-T_end_ interval by 0.16 (95% CI −0.25–0.58; *p* = 0.45; I^2^ = 95%; *p* < 0.01; Figure 2). However, standardized mean difference did not differ when comparing both subgroups (*p* = 0.17).

**Figure 3:**
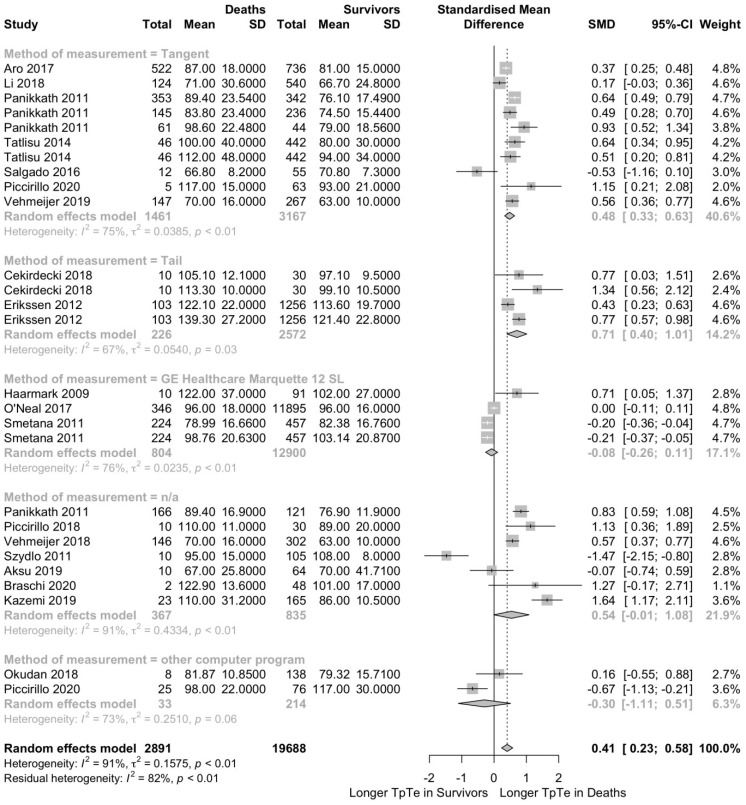
T_peak_-T_end_ interval is longer in patients who died compared to survivors. This forest plot presents the same studies as Figure 2 but categorized by the ECG measuring method to determine the T_peak_-T_end_ interval [25,27,29,30,31,32,34,35,37,38,39,40,41,42,43,46,49,50,51,52,53].

A second subgroup analysis was conducted to determine the effect of the measurement method. All-cause deaths had a longer T_peak_-T_end_ interval in subgroups reporting the tangent method (SMD 0.48; 95% CI 0.33–0.63; *p* < 0.001; I^2^ = 75%; *p* < 0.01; Figure 3) and the tail method (SMD 0.71; 95% CI 0.40–1.01; *p* < 0.001; I^2^ = 67%; *p* = 0.03; Figure 3). This finding did not apply for subgroups using the GE Healthcare Marquette 12SL algorithm (SMD −0.08; 95% CI −0.26–0.11; *p* = 0.42; I^2^ = 76%; *p* < 0.01; Figure 3) or other computer programs (SMD −0.30; 95% CI −1.11–0.51; *p* = 0.47; I^2^ = 73%; *p* < 0.06; Figure 3) and in the subgroup without specification of the measurement method (SMD 0.54; 95% CI −0.01–1.08; *p* = 0.05; I^2^ = 91%; *p* < 0.01; Figure 3). 

To account for possible bias by study design or study quality, we conducted two post-hoc sensitivity analyses. Both a sensitivity analysis restricted to 11 studies with a prospective cohort study design including 17,063 patients, and a sensitivity analysis restricted to 12 studies with a Newcastle-Ottawa Quality Assessment Scale score greater six, including 19,842 patients, confirmed the finding. In both prospective cohort studies and studies with a Newcastle-Ottawa Quality Assessment Scale score greater than six, all-cause deaths had a longer T_peak_-T_end_ interval of 0.38 (95% CI 0.08–0.67; *p* = 0.0141; I^2^ = 92 %, *p* < 0.001; Figure S1) and 0.50 (95% CI 0.33–0.67; *p* < 0.001; I^2^ = 87%, *p* < 0.01; Figure S2), respectively. 

### 3.3. Unadjusted Odds of Death in Patients with Longer vs. Shorter T_peak_-T_end_ Intervals

**Figure 4:**
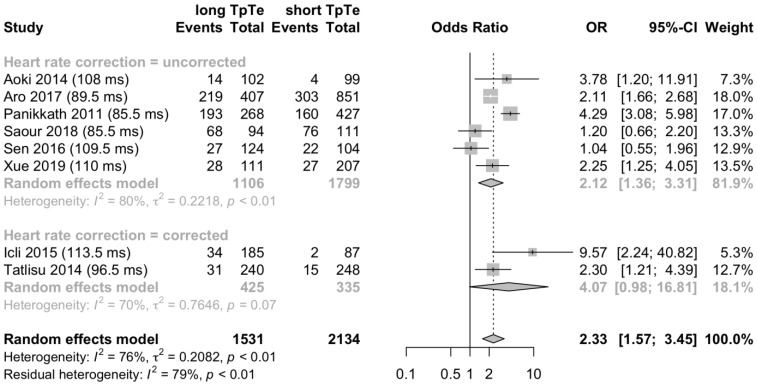
A long T_peak_-T_end_ interval is associated with all-cause death. The forest plot shows the data from 3665 patients from eight studies, comparing long and short T_peak_-T_end_ interval in deaths and the total number of patients with the respective T_peak_-T_end_ interval. Classification in long and short T_peak_-T_end_ interval was adopted from studies according to the reported cut-off value. A long T_peak_-T_end_ interval is associated with death by an odds ratio (OR) of 2.33. TpTe = T_peak_-T_end_ interval; OR = odds ratio; CI = confidence interval [26,27,33,39,47,48,51,54].

A fourfold table with long and short T_peak_-T_end_ interval versus all-cause deaths and survivors was available for eight patient populations in seven articles and one conference abstract, reporting data for 3665 patients and 1223 all-cause deaths. We contacted 11 authors for additional outcome data for this analysis and received the requested data from one author. The most frequent patient population analyzed was heart failure. The random effects model for this analysis is presented in Figure 4. A combined analysis revealed an overall odds ratio of 2.33 (95% CI 1.57–3.45; *p* < 0.001; I^2^ = 76%; *p* < 0.01); indicating that a long T_peak_-T_end_ interval was significantly associated with all-cause death.

A subgroup analysis was performed comparing six studies reporting mortality data associated with an uncorrected T_peak_-T_end_ interval and two studies reporting mortality data associated with a heart rate-corrected T_peak_-T_end_ interval. The subgroups included 2905 patients and 760 patients, respectively. In the pooled analysis, in both subgroups, a long T_peak_-T_end_ interval was associated with an increased risk of all-cause death: A long uncorrected T_peak_-T_end_ interval was associated with all-cause death by an odds ratio of 2.12 (95% CI 1.36–3.31; *p* < 0.01; I^2^ = 80%; *p* < 0.01; Figure 4) and in tendency long heart rate-corrected T_peak_-T_end_ interval by 4.07 (95% CI 0.98–16.81; *p* = 0.05 I^2^ = 70%; *p* = 0.07; Figure 4). However, standardized mean difference did not differ when comparing both subgroups (*p* = 0.39).

**Figure 5:**
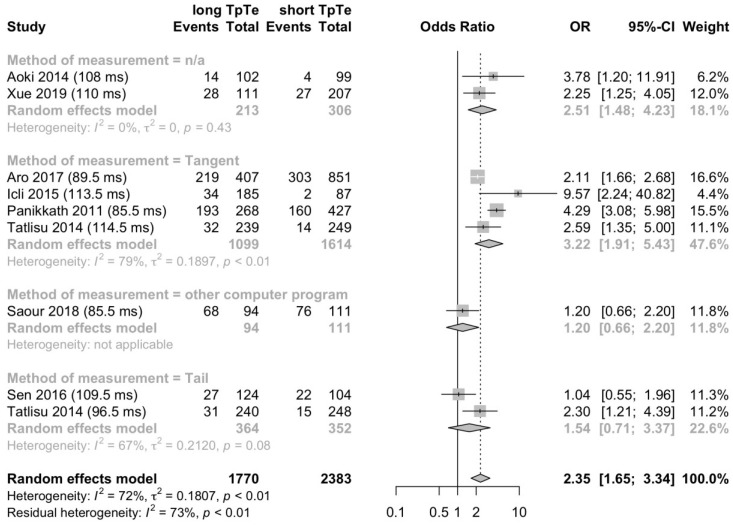
A long T_peak_-T_end_ interval is associated with all-cause death. This forest plot presents the same studies as Figure 4 but categorized by the ECG measuring method to determine the T_peak_-T_end_ interval [26,27,33,39,47,48,51,54].

A second subgroup analysis was conducted to determine the effect of the measurement method. A long T_peak_-T_end_ interval was associated with all-cause death in the subgroup reporting the tangent method (OR 3.22; 95% CI 1.91–5.43; *p* < 0.001; I^2^ = 79%; *p* < 0.01; Figure 5) and in the subgroup without specification of the measurement method (OR 2.51; 95% CI 1.48–4.23; *p* < 0.01; I^2^ = 0%; *p* = 0.43; Figure 5). This finding did not apply for subgroups reporting the tail method (OR 1.54; 95% CI 0.71–3.37; *p* = 0.28; I^2^ = 67%; *p* = 0.08; Figure 5) and other computing programs (OR 1.20; 95% CI 0.66–2.20; Figure 5). 

To account for possible bias by study design or study quality, we conducted two post-hoc sensitivity analyses. Both a sensitivity analyses restricted to four studies with a prospective cohort study design including 1235 patients, and a sensitivity analyses restricted to six studies with a Newcastle-Ottawa Quality Assessment Scale score greater six, including 3146 patients, confirmed this finding. In both prospective cohort studies and studies with a Newcastle-Ottawa Quality Assessment Scale score greater than six, a long T_peak_-T_end_ interval was associated with an increased risk of all-cause death of 1.95 (95% CI 1.20–3.15; *p* = 0.0066; I^2^ = 46%; *p* = 0.14; Figure S3) and 2.25 (95% CI 1.39–3.65; *p* = 0.0010;I^2^ = 82%; *p* < 0.01; Figure S4), respectively. 

### 3.4. Predicting Death with T_peak_-T_end_ Interval 

A total of six studies including 3951 patients reported AUCs for receiver operating characteristics (ROC) with corresponding cutoffs, sensitivity and specificity, analyzing predictability of all-cause death on the basis of the T_peak_-T_end_ interval (Table 2). 

**Figure 6:**
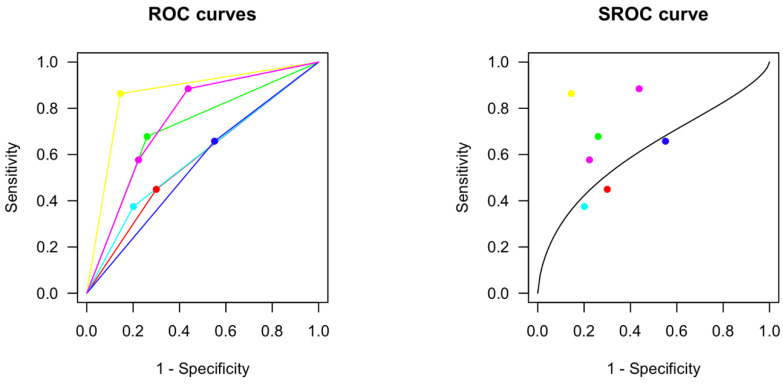
Estimated receiver operating characteristics of the seven reported AUROCS with corresponding sensitivity and specificity (**left**) and the estimated summary ROC (SROC) curve with an AUC of 0.65 (**right**). (Bombelli (2016) [28] (red); Cekridecki (2018) [30] (yellow); Erikssen (2012) [31] (green); Morin (2012) [36] (light blue); Rosenthal (2015) [45] (dark blue); Salgado (2016) [46] (pink)).

One study reported AUROC, sensitivity and specificity for two different cutoffs in the same population. Meta-analysis of these studies estimated a summary ROC curve with an AUC of 0.65 and estimated an optimal cutoff at 98.8 ms (Figure 6). At the optimal cutoff, sensitivity would be 0.61 (95%CI 0.37–0.81) and specificity 0.61 (95%CI 0.41–0.77).

### 3.5. Secondary Endpoints

No study reported the predefined secondary endpoints increase in troponin and NSTEMI with regard to the T_peak_-T_end_ interval. During full-text review, several studies were identified as likely to have these data but none of the three authors contacted for data on increase in troponin and one contacted for NSTEMI responded to our request.

## 4. Discussion

In this meta-analysis, we analyzed 29 studies to determine the association of the T_peak_-T_end_ interval with mortality outcomes. There is strong evidence of an association considering our main findings. All-cause deaths had longer T_peak_-T_end_ intervals compared to survivors by a standardized mean difference of 0.41 (95% CI 0.23–0.58) and patients with a long T_peak_-T_end_ interval had a higher risk of all-cause death compared to patients with a short T_peak_-T_end_ interval by an overall odds ratio of 2.33 (95% CI 1.57–3.45). Our subgroup analyses also suggest that heart rate-correction of the T_peak_-T_end_ interval did not affect the association of T_peak_-T_end_ interval with mortality outcomes, whereas this finding did only apply to the two most common ECG methods of measurement, the tangent and the tail method. In a summary AUROC analysis based on studies analyzing the predictability of the T_peak_-T_end_ interval for all-cause mortality, we found a summary AUROC of 0.65 and an optimal cutoff at 99 ms. 

Considering that 21 studies reported the duration of the T_peak_-T_end_ interval for all-cause deaths and survivors, while eight studies reported the number of all-cause deaths and survivors with short and long T_peak_-T_end_ interval based on their respective cutoff, we performed two different analyses to include all available data on the association of the T_peak_-T_end_ interval with all-cause death. Both calculated summary statistics indicated a significant association and, therefore, strengthen the results of our meta-analysis. 

We know of one former meta-analysis investigating the T_peak_-T_end_ interval in different patient populations with regard to mortality outcomes [10]. Since then, 14 studies have been published and the inclusion of these is keeping our meta-analysis up to date. Similar to Tse et al., we included studies regardless of the patient population and, therefore, accepted a wide variation in study populations. While Tse et al. found higher T_peak_-T_end_ intervals in a subgroup of three studies to be associated with 4.5 higher odds of all-cause death, our analysis showed higher odds of 2.3 in eight more recent studies. However, the exclusion of patients with Brugada syndrome most likely reduced bias in our results. Brugada syndrome, characterized by depolarization and repolarization heterogeneities [55,56], is predisposing particularly symptomatic patients to ventricular tachycardia and fibrillation and sudden cardiac death [57,58]. However, other covariates besides the congenital dispersion of repolarization, such as the occurrence of a syncope [59], might impact the likelihood of developing adverse events and bias the association of the T_peak_-T_end_ interval and all-cause death. 

Further sources of heterogeneity of the included studies were present in our meta-analysis including different study designs, methods and leads of ECG measurement or whether a heart rate-correction was implemented. Based on the a priori assumption of these limitations and their discussion following the publication of the former meta-analysis [11], we conducted the desired subgroup analyses on the method of measurement and heart rate-correction. Unfortunately, high variability in the ECG leads used for measuring the T_peak_-T_end_ interval did not allow for a subgroup analysis of this feature, instead we analyzed the standardized mean difference to account for this heterogeneity. To account for possible introduction of bias by study design (prospective vs. retrospective) or study quality, we conducted two sensitivity analyses. All sensitivity analyses, including only prospective cohort studies or including only high-quality studies confirmed our results and rendered the introduction of bias to our results by study design or study quality unlikely.

The T_peak_-T_end_ interval can be manually measured using two methods. For the ‘tangent method’, it is defined as the time from the peak of the T wave and the intersection between the tangent at the steepest point of the T-wave downslope and the isoelectric line [60]. For the ‘tail method’, it is defined as the time from the peak of the T wave to the point where the wave reaches the isoelectric line [61]. Both definitions could be found repeatedly in various studies, but there is no agreement on the optimal method of measurement. Tatlisu et al. found the tail method to be a better predictor of mortality in STEMI patients compared to the tangent method [51], while Rosenthal et al. found both methods to be similarly predictive for mortality and rather recommend an automated measurement method, such as the GE Healthcare Marquette 12 SL/QT Guard plus analysis program (12 SL), considering its higher reliability and reproducibility [62]. The measurement of high-precision digital ECGs could possibly narrow measurement inaccuracies and measurement heterogeneity in the future [12]. Additionally, a consistency regarding the measurement of more complex T wave morphologies still needs to be established.

The heart rate dependence of the T_peak_-T_end_ interval is controversial. Increasing heart rate is principally known to shorten the QT interval [63]. However, previous analyses suggested the T_peak_-T_end_ interval to be only minimally or even not at all dependent on the heart rate [64,65]. Several of the included studies in this meta-analysis still performed a heart rate-correction mainly using the method described by Bazett and colleagues [66]. At high heart rates, the Bazett formula overcorrects the QT interval and leads to artificially prolonged QTc intervals [63]. Therefore, the heart rate-correction by Fridericia and colleagues [67] has become the preferable method by now [63,68,69]. However, all approaches using a formula are subject to limitations since their derivation is bound to specific patient populations and the dependence of the QT interval on heart rate can be altered by different diseases and drugs [63]. An alternative approach is to determine the heart rate dependence for each individual whilst taking into account biological differences [63,68,70,71]. Future studies will possibly clarify the heart rate dependence of the T_peak_-T_end_ interval and examine the feasibility of subject-specific correction for routine clinical analysis. 

On the basis of included studies analyzing the predictability of the T_peak_-T_end_ interval, the T_peak_-T_end_ interval can be used as a prognostic tool to a limited extent only. Very few studies provided AUROC data and the available data was highly heterogeneous. Heterogeneity in the predictive accuracy was possibly due to differences in the study populations, methodological approaches and outcome measures. The analysis of AUROC data would potentially be more promising regarding the prediction of NSTEMI and troponin increase on the basis of the T_peak_-T_end_ interval. Compared to death, both entities are rather direct consequences to repolarization disorders and arrhythmogenic events, possibly increasing the predictive value of future AUROC analyses.

This meta-analysis presents a great body of evidence supporting the association between the T_peak_-T_end_ interval and all-cause death and confirms the value of the T_peak_-T_end_ interval for risk stratification. Considering that the included data was very heterogeneous in several aspects and consistency in the measurement of the T_peak_-T_end_ interval is still not established, further studies are needed to confirm our summary findings. 

The QT interval is subject to beat-to-beat variability and heart rate changes [63,72]. Especially in patients with atrial fibrillation and bundle branch block, the QT interval varies greatly based on different cycle lengths [73,74]. However, the results of our meta-analysis indicate that the predictability of all-cause death is lower when the T_peak_-T_end_ interval is corrected for heart rate. This might support the aforementioned consideration of the T_peak_-T_end_ interval as a heart rate-independent and steady marker of repolarization disorders. A prospective longitudinal analysis is desirable to clarify the heart rate dependence of the T_peak_-T_end_ interval. To account for the respiration-induced beat-to-beat variability of the T_peak_-T_end_ interval, a measurement of an averaged T_peak_-T_end_ interval from a large number of consecutive beats could be implemented [11]. 

All-cause death as the primary endpoint of this meta-analysis included fulminant courses only. However, NSTEMI and troponin elevation and their association with the T_peak_-T_end_ interval as a surrogate marker for repolarization disorders are also of great interest. We assumed to obtain enough data for a secondary endpoint analysis, but so far this association has been investigated barely to not at all. We are currently planning a retrospective analysis of several thousand pre-clinical ECGs from patients with acute coronary syndrome in order to evaluate the association between the T_peak_-T_end_ interval and the incidence of NSTEMI or troponin increase. Moreover, the change of the T_peak_-T_end_ interval over time could also be of great interest for future clinical analysis. The association of the T_peak_-T_end_ interval and delta troponin or decreasing troponin after reperfusion therapy might be an interesting addition for future studies. 

In conclusion, all-cause deaths had longer T_peak_-T_end_ intervals compared to survivors and patients with a long T_peak_-T_end_ interval had a higher risk of all-cause death compared to patients with a short T_peak_-T_end_ interval. Based on the few studies analyzing the predictability of the T_peak_-T_end_ interval, the prognostic value of the T_peak_-T_end_ interval is limited. Consequently, further large-scale prospective and consistently performed studies are required.

## Figures and Tables

**Table 1:** Study characteristics.

First Author Year	Location	Study Design	Pathology	Sample Size (*n*)	Women (*n*, [%])	Age (Years)	Leads for Measuring T_peak_-T_end_ Interval	Method for Measuring T_peak_-T_end_ Interval	Heart-Rate Correction of T_peak_-T_end_ Interval?	Follow-Up Duration (Months)	Endpoint
Aksu, E. 2019 [25]	Turkey	Retrospective cohort study *	Chronic hemodialysis patients	74	37 (50)	n/a	n/a	n/a	Uncorrected	12–15	Cardiac death
Aoki, S. 2014 [26]	Japan	Prospective cohort study *	Acute heart failure syndrome	201	115 (57.2)	78.4 ± n/a	n/a	n/a	Uncorrected	161 ± n/a	Cardiac death
Aro, A. L. 2017 [27]	USA, Finland	Case control study	SCD	1258	409 (32.5)	65.6 ± 12.8 ^a^	V5	Tangent method	Uncorrected	n/a	SCD
Bombelli, M. 2016 [28]	Italy	Cross-sectional study	New onset hypertension	1853	928 (50)	50.4 ± 13.5	V5	Tail method	Heart-rate- corrected	192 ± n/a	All-cause mortality + cardiovascular mortality
Braschi, A. 2020 [29]	Italy	Retrospective cohort study	Patients with Takotsubo syndome	50	n/a	66.2 ± 9.9	Precordial lead with the longest T_peak_-T_end_ interval	n/a	Uncorrected	n/a (during hospitalization)	All-cause mortality
Cekirdecki, E. I. 2019 [30]	Turkey	Retrospective cohort study	Arrhythmo-genic right ventricular cardiomyo-pathy	40 ^d^	10 (25)	34.0 ± 11.53 ^c^	II, V2, V5 (lead with the longest T_peak_-T_end_)	Tail method	Heart-rate- corrected + uncorrected	9.5 ± 30.5	All-cause mortality
Erikssen, G. 2012 [31]	Norway	Prospective cohort study	STEMI and NSTEMI patients who underwent PCI	1359 ^e^	839 (61.7)	65.5 ± 12.5 ^a^	Precordial lead with the longest T_peak_-T_end_ interval	Tail method	Heart-rate- corrected + uncorrected	15.6 ± n/a	All-cause mortality
Haarmark, C. 2009 [32]	Denmark	Prospective cohort study	Patients with STEMI undergoing PCI	101	27 (26.7)	62 ± n/a	Non-infarct-related leads (ST-segment deviations below 0.055 mV at the J-point in the pre-PCI ECG) V5, V4, V6, II, III, and I (in descending order)	GE Healthcare Marquette 12SL	Uncorrected	22.5 ± 6.9	All-cause mortality
Icli, A. 2015 [33]	Turkey	Retrospective cohort study	Acute pulmonary embolism	272	119 (43.8)	63.1 ± 16.8	V5 or, if V5 was not suitable, V4 and V6 in that order were used	Tangent method	Corrected	1.0 ± n/a	All-cause mortality
Kazemi, B. 2019 [34]	Iran	Prospective cohort study	STEMI patients undergoing primary PCI or thrombo-lytic therapy	188	116 (61.7)	85.97 ± 9.93	Leads without ST-segment elevation	n/a	Uncorrected	n/a (hospitalization period)	Cardiac death
Li, J. 2019 [35]	Switzerland	Nested case control study	Patients after PCI	644	152 (23.6)	68.5 ± 12.3	II or V5 when a parameter was not measurable in lead II	Tangent method	Uncorrected	n/a (within 1 year)	All-cause mortality
Morin, D. P. 2012 [36]	USA	Retrospective cohort study	Patients with an implanted ICD and LVEF ≤ 35%	327	83 (25.4)	67 ± 11	V2-V5	GE Healthcare Marquette 12SL	Heart-rate- corrected + uncorrected	30 ± 13	All-cause mortality
Okudan, Y. E. 2018 [37]	Turkey	Retrospective cohort study *	Patients with acute anterior MI	146	n/a	n/a	Precordial leads	BitRule programme	Uncorrected	n/a (first month and first year MACE)	Cardiovascular mortality
O’Neal, W. T. 2017 [38]	USA	Prospective cohort study	General Population (participants from ARIC study)	12,241	6781 (55.4)	54 ± 5.7	Median value of all 12 leads	GE Healthcare Marquette 12SL	Heart-rate-corrected	273.6 ± 34.8 ^c^	SCD
Panikkath, R. 2011 [39]	USA	Case control study	SCD from out-of-hospital cardiac arrests	695	116 (16.7)	66.6 ± 14.4	V5 or, if this lead was not suitable, leads V4 and V6 in that order were used	Tangent method	Uncorrected	n/a	SCD
			SCD with normal QTc		n/a	n/a					
			SCD with intraventri-cular conduction delay		17 (2.4)	72.3 ± 13.9					
Panikkath, R. 2011 [40]	USA	Nested case-control study *	QT prolonging drugs	287	93 (32.4)	65.5 ± 13.3 ^a^	V5	n/a	Uncorrected	n/a	SCD
Piccirillo, G. 2018 [41]	Italy	Prosepctive cohort study	Transcatheter aortic valve replacement patients	40	17 (42.5)	81.0 ± 7.0	n/a	n/a	Uncorrected	12.0 ± n/a	All-cause mortality + cardiovascular mortality
Piccirillo, G. 2020 [42]	Italy	Prospective cohort study	Low SCD risk-out-patients with asymp-tomatic and treated car-diovascular risk factors - elderly subgroup (>60 years)	68	40 (58.8)	73.82 ± 7.26	n/a	Tangent method	Uncorrected	27.6 ± 6	All-cause mortality
Piccirillo, G. 2020 [43]	Italy	Prospective cohort study	Patients with decompensated CHF	101	47 (46.5)	83 ± 11 ^a^	n/a	Software by Berger et al.	Uncorrected	n/a (during hospitalization)	All-cause mortality
Piccirillo, G. ^f^ 2020 [44]	Italy	Prospective cohort study	Patients with decompensated CHF	113	54 (47.8)	82.7 ± 10.3	n/a	Software by Berger et al.	Uncorrected	1 ± n/a	All-cause mortality
Rosenthal, T. M. 2015 [45]	USA	Prospective cohort study	Systolic cardiomyopathy (Patients with an implanted ICD and LVEF ≤ 35%)	305	82 (26.9)	70 ± 11	V2-V5 (values of T_peak_ are averaged to obtain global T_peak_)	GE Healthcare Marquette 12SL	Heart-rate- corrected + uncorrected	49 ± 21	All-cause mortality
Salgado, A. A. 2016 [46]	Brazil	Prospective cohort study	Liver Cirrhosis	67	32 (47.8)	54.0 ± 1.9 ^a^	All leads	Tangent method	Uncorrected	9.7 ± 6.8 ^c^	All-cause mortality
Saour, B. M. 2019 [47]	USA	Retrospective cohort study	End stage renal disease	205	1 (0.5)	66.6 ± 12.3	V5 or, if V5 was not inter-pretable, V4 and then V6 were used	Difference of QT interval and QRS complex	Uncorrected	42 ± n/a	All-cause mortality + SCD + Non-SCD
Sen, Ö. 2016 [48]	Turkey	Prospective cohort study	Heart failure patients undergoing ICD implantation	228	56 (24.6)	59.3 ± 12.3	Precordial lead with the longest T_peak_-T_end_ interval	Tail method	Uncorrected	22.3 ± 7.7	All-cause mortality
Smetana, P. 2011 [49]	England, Austria	Retrospective cohort study	Male US veterans with cardio-vascular disease	681	0 (0)	61.05 ± 10.25	V4–V6	GE Healthcare Marquette 12SL	Heart-rate- corrected + uncorrected	87.6 ± 44.4	All-cause mortality
Szydlo, K. 2011 [50]	Poland	Prospective cohort study *	Patients with anterior MI treated with primary PCI	115	28 (24.3)	58.43 ± 11.21 ^a^	n/a	n/a	Heart-rate-corrected	n/a (within 36 months)	Cardiac death
Tatlisu, M. A. 2014 [51]	Turkey	Prospective cohort study	STEMI undergoing primary PCI	488	79 (16.2)	55.6 ± 11.2 ^a^	Leads without ST-segment elevation; the longest T_peak_-T_end_ interval was chosen	Tail method/ Tangent method	Heart-rate- corrected + uncorrected	21 ± 10.2	All-cause mortality
Vehmeijer, J. T. 2018 [52]	Netherlands	Prospective cohort study *	Adults with congenital heart disease	448	152 (33.9) ^b^	35.9 ± 16.2 ^b,c^	One T-wave of each ECG lead	n/a	Uncorrected	n/a	SCD
Vehmeijer, J. T. 2019 [53]	Netherlands	Case control study	Adults with congenital heart disease	414	147 (35.5)	35.9 ± 15.3 ^a,c^	One T-wave of each ECG lead	Tangent method	Uncorrected	n/a	SCD
Xue, C. 2019 [54]	China	Prospective cohort study	Heart failure patients with an implantable cardioverter-defibrillator	318	79 (24.8)	57.59 ± 11.36	Median value of all 12 leads	n/a	Uncorrected	32.12 ± 25.07	All-cause mortality

^a^ According to the method by Altman et al. (2000): Combined means and standard deviations of two study populations into one group. ^b^ According to the method by Altman et al. (2000): Assumed combined statistics because cases and controls were matched for age and sex. ^c^ According to the method by Wan et al. (2014): Estimated means and standard deviations from sample size, median and interquartile range. ^d^ We included 40 patients of the entire population of 105 patients, because mortality outcomes were reported for this subgroup only. ^e^ We included 1359 patients of the entire population of 1384 patients, because ECG data was available for this subgroup only. ^f^ This study was excluded from the analysis, because the study population overlaps to a large extent with the study listed above. * conference abstract; n/a: not applicable; SCD: sudden cardiac death; STEMI: ST elevation myocardial infarction; NSTEMI: non-ST-elevation myocardial infarction; PCI: percutaneous coronary intervention; ICD: implanted cardiac defibrillator; LVEF: left ventricular ejection fraction; MACE: major adverse cardiovascular events; CHF: chronic heart failure.

**Table 2:** List of studies reporting area under the receiver operating characteristics (AUROC) curves to predict all-cause death by Tpeak-Tend interval.

Study (Year)	Cutoff (ms)	AUROC	Sensitivity	Specificity
Bombelli (2016) [28]	121	0.59	0.45	0.70
Cekridecki (2018) [30]	107	0.89	0.90	0.88
Erikssen (2012) [31]	132	0.77	0.68	0.74
Morin (2012) [36]	126,7	0.60	0.37	0.80
Rosenthal (2015) [45]	104	0.58	0.66	0.45
Salgado (2016) [46]	50	0.69	0.90	0.57
Salgado (2016) [46]	60	0.76	0.60	0.79

## Data Availability

The data presented in this study are available in the Figures and Tables presented in this article and the Supplementary Materials.

## Supplementary Materials

The following supporting information can be downloaded at: https://www.mdpi.com/article/10.3390/jcm12030992/s1, Resource S1: Display of the full electronic search strategy for MEDLINE via PubMed, Table S1: Quality Assessment of the included Studies, Resource S2: Detailed Quality Assessment Method used for Table S1, Figure S1: T_peak_-T_end_ interval is longer in patients who died compared to survivors in prospective cohort studies; Figure S2: T_peak_-T_end_ interval is longer in patients who died compared to survivors in studies with a Newcastle-Ottawa-Scale greater than six. Figure S3: A long T_peak_-T_end_ interval is associated with all-cause in-hospital death in prospectice cohort studies only. Figure S4: A long T_peak_-T_end_ interval is associated with all-cause in-hospital death in studies with an Newcastle-Ottawa-Scale greater than 6.
