# Supplementary material for: A Longer Tpeak-Tend Interval Is Associated with a Higher Risk of Death: A Meta-Analysis
Source: J Clin Med. 2023 Jan 28;12(3):992. doi: 10.3390/jcm12030992 (PMC9917475; doi:10.3390/jcm12030992)

Supplementary Material

A Longer T_{peak}-T_{end} Interval Is Associated with a Higher Risk of Death: A Meta-Analysis

Cathrin Caroline Braun [1], Matthias Daniel Zink [2], Sophie Gozdowsky [3], Julie Martha Hoffmann [1], Nadine Hochhausen [1], Anna Bettina Röhl [1], Stefan Kurt Beckers [1,4] and Felix Kork [1,*]

[1]Department of Anesthesiology, Medical Faculty, RWTH Aachen University, Pauwelsstraße 30, 52074 Aachen, Germany

[2]Department of Cardiology, Medical Faculty, RWTH Aachen University, Pauwelsstraße 30, 52074 Aachen, Germany

[3]Medical Management, Emergency Medical Service, Berlin Fire Brigade, 10150 Berlin, Germany

[4]Aachen Institute of Emergency Medicine and Civil Security, Medical Faculty, RWTH Aachen University, Pauwelsstraße 30, 52074 Aachen, Germany

Corresponding author:

Priv.-Doz. Dr. med. Felix Kork, M.Sc.

Medical Faculty, RTWH Aachen University

Department of Anesthesiology

Pauwelsstraße 30

52074 Aachen

E-mail: fkork@ukaachen.de

Fon:	+49 241 80 88179

Fax:	+49 241 80 82406

Online Resource S1. Display of the full electronic search strategy for MEDLINE via PubMed

((((((((((((((Tpeak - Tend[Title/Abstract]) OR (Tpeak-Tend[Title/Abstract])) OR (Tp - Te[Title/Abstract])) OR (Tp-Te[Title/Abstract])) OR (Tpeak-end[Title/Abstract])) OR (Tp-e[Title/Abstract])) OR (T(peak)-T(end)[Title/Abstract])) OR (T wave peak-to-end[Title/Abstract])) OR (T peak-T end[Title/Abstract])) OR (TPEc[Title/Abstract])) OR (T-peak to T-end[Title/Abstract])) OR (Tpeak-to-Tend[Title/Abstract])) OR (T-Wave Peak to T-Wave End[Title/Abstract])) OR (TpTe[Title/Abstract])) AND (("2008/01/01"[Date - Publication] : "3000"[Date - Publication]))

Online Table S1. Quality Assessment of the included studies

Newcastle-Ottawa Quality Assessment Scale for Cohort Studies

Studies	**Selection** (maximum of four stars)				**Comparability** (maximum of two stars)	**Outcome** (maximum of three stars)			**Total Score** (0-9 stars)
	Representa-tiveness of the exposed cohort[a]	Selection of the non-exposed cohort[a]	Ascertainment of exposure	Demonstration that the outcome of interest was not present at start of study	Comparability of cohorts on the basis of the design or analysis	Assessment of outcome	Demonstration that the follow-up was long enough for outcomes to occur	Adequacy of follow up cohorts	
Aksu et al., 2019 [25]	❈	❈	❈	❈			❈		5
Aoki et al., 2014 [26]	❈	❈	❈	❈			❈		5
Braschi et al., 2020 [29]	❈	❈	❈	❈	❈❈				6
Cekirdecki et al., 2018 [30]	❈	❈	❈	❈	❈❈	❈	❈		8
Erikssen et al., 2012 [31]	❈	❈	❈	❈	❈❈	❈	❈	❈	9
Haarmark et al., 2009 [32]	❈	❈	❈	❈		❈	❈	❈	7
Icli et al., 2015 [33]	❈	❈	❈	❈	❈❈		❈	❈	8
Kazemi et al., 2019 [34]	❈	❈	❈	❈		❈			5
Morin et al., 2012 [36]	❈	❈	❈	❈	❈❈	❈	❈		8
Okudan et al., 2018 [37]	❈	❈	❈	❈			❈		5

O’Neal et al., 2017 [38]	❋	❋	❋	❋	❋❋	❋	❋		8
Piccirillo et al., 2018 [41]	❋	❋	❋	❋	❋❋		❋		7
Piccirillo et al., 2020 [42]	❋	❋	❋	❋	❋❋		❋		7
Piccirillo et al., 2020 [43]	❋	❋	❋	❋	❋❋	❋			7
Rosenthal et al., 2017 [45]	❋	❋	❋	❋	❋❋	❋	❋		8
Salgado et al., 2016 [46]	❋	❋	❋	❋			❋		5
Saour et al., 2018 [47]	❋	❋	❋	❋	❋[b]	❋	❋		7
Sen et al., 2016 [48]	❋	❋	❋	❋	❋❋		❋		7
Smetana et al., 2011 [49]		❋	❋	❋	❋[b]	❋	❋		6
Szydlo et al., 2011 [50]	❋	❋	❋	❋			❋		5
Tatlisu et al., 2014 [51]	❋	❋	❋	❋	❋❋		❋	❋	8
Vehmeijer et al., 2018 [52]	❋	❋	❋	❋	❋[b]				5
Xue et al., 2019 [54]	❋	❋	❋	❋			❋	❋	6

[a]For this item we assessed patients with long Tpeak-Tend interval vs. normal Tpeak-Tend interval; if the data we extracted was from a subgroup of the study population, we assessed the item for this subgroup. [b]This study did not control for age and sex, but it controlled for other factors; for detailed quality assessment method please refer to Online Ressource S2.

Newcastle-Ottawa Quality Assessment Scale for Case Control Studies

Studies	Selection (maximum of four stars)				Comparability (maximum of two stars)	Exposure (maximum of two stars)		Total Score (0-8 stars)
	Is the case definition adequate?	Representativeness of the cases	Selection of controls	Definition of controls	Comparability of cases and controls on the basis of the design or analysis	Ascertainment of exposure	Same method of ascertain-ment for cases and controls	
Aro et al., 2017 [27]	✵	✵	✵	✵	✵✵	✵	✵	8
Li et al., 2018 [35]		✵	✵	✵	✵✵	✵	✵	7
Panikkath et al., 2011 [39]	✵	✵	✵	✵	✵✵	✵	✵	8
Panikkath et al., 2011 [40]			✵	✵	✵	✵	✵	5
Vehmeijer et al., 2019 [53]	✵	✵	✵	✵	✵✵	✵	✵	8

For detailed quality assessment method please refer to Online Ressource S2.

Newcastle-Ottawa Quality Assessment Scale adapted for Cross Sectional Studies

Studies	Selection (maximum of five stars)				Comparability (maximum of two stars)	Outcome (maximum of three stars)		Total Score (0-8 stars)
	Representativeness of the sample	Sample size	Non-respondents	Ascertainment of exposure	Comparability of subjects in different outcome groups on the basis of the design or analysis. Confounding factors are controlled.	Assessment of outcome	Statistical test	
Bombelli et al., 2016 [28]	✵	✵		✵	✵✵		✵	6

For detailed quality assessment method please refer to Online Ressource S2.

Online Ressource S2: Detailed quality assessment method used for Online Table S1.

NEWCASTLE - OTTAWA QUALITY ASSESSMENT SCALE
COHORT STUDIES

Note: A study can be awarded a maximum of one star for each numbered item within the Selection and Outcome categories. A maximum of two stars can be given for Comparability

Selection

1) Representativeness of the exposed cohort:
   a) truly representative of the average patient, that the respective study sought to investigate in the community ❋ (■)
   b) somewhat representative of the average patient, that the respective study sought to investigate in the community ❋ (■)
   c) selected group of users e.g. nurses, volunteers (■)
   d) no description of the derivation of the cohort (■)

2) Selection of the non exposed cohort:
   a) drawn from the same community as the exposed cohort ❋ (■)
   b) drawn from a different source (■)
   c) no description of the derivation of the non exposed cohort (■)

3) Ascertainment of exposure:
   a) secure record (e.g. surgical records) ❋ (■)
   b) structured interview ❋ (■)
   c) written self report (■)
   d) no description (■)

4) Demonstration that outcome of interest was not present at start of study:
   a) yes ❋ (■)
   b) no (■)

Comparability

1) Comparability of cohorts on the basis of the design or analysis:
   a) study controls for age and sex ❋ (■)
   b) study controls for any additional factor ❋ (■)
   c) No description (■)

Outcome

1) Assessment of outcome:
   a) independent blind assessment ❋ (■)
   b) record linkage ❋ (■)
   c) self report (■)
   d) no description (■)

2) Was follow-up long enough for outcomes to occur:
   a) yes (mortality follow-up of at least 30 days or troponin assay not exceeding 48h) ❋ (■)
   b) no (■)

3) Adequacy of follow up of cohorts:
   a) complete follow up - all subjects accounted for ❋ (■)
   b) subjects lost to follow up unlikely to introduce bias - small number lost - > 90 % follow up, or description provided of those lost ❋ (■)
   c) follow up rate < 90 % and no description of those lost (■)
   d) no statement (■)

NEWCASTLE - OTTAWA QUALITY ASSESSMENT SCALE
CASE CONTROL STUDIES

Note: A study can be awarded a maximum of one star for each numbered item within the Selection and Exposure categories. A maximum of two stars can be given for Comparability.

Selection

1) Is the case definition adequate?
   a) yes, with independent validation ❋ (■)
   b) yes, e.g. record linkage or based on self reports (■)
   c) no description (■)

2) Representativeness of the cases:
   a) consecutive or obviously representative series of cases ❋ (■)
   b) potential for selection biases or not stated (■)

3) Selection of Controls:
   a) community controls ❋ (■)
   b) hospital controls (■)
   c) no description (■)

4) Definition of Controls:
   a) no history of disease (endpoint) ❋ (■)
   b) no description of source (■)

Comparability

1) Comparability of cases and controls on the basis of the design or analysis:
   a) study controls for age and sex ❋ (■)
   b) study controls for any additional factor (■)
   c) No description (■)

Exposure

1) Ascertainment of exposure:
   a) secure record (e.g. surgical records) ❋ (■)
   b) structured interview where blind to case/control status ❋ (■)
   c) interview not blinded to case/control status (■)
   d) written self report or medical record only (■)
   e) no description (■)

2) Same method of ascertainment for cases and controls:
   a) yes ❋ (■)
   b) no (■)

3) Non-Response rate:
   a) same rate for both groups ❋
   b) non respondents described
   c) rate different and no designation

Not applicable because we did not analyze studies using questionnaires; the case control studies we included, investigated sub-populations from existing registries or prospective cohort studies

NEWCASTLE - OTTAWA QUALITY ASSESSMENT SCALE
ADAPTED FOR CROSS SECTIONAL STUDIES

Selection (maximum of 5 stars)

1) Representativeness of the sample:
   a) truly representative of the average patient, that the respective study sought to investigate in the target population ❋ (■)
   b) somewhat representative of the average patient, that the respective study sought to investigate in the target population ❋ (■)
   c) selected group of users (■)
   d) no description of the sampling strategy (■)

2) Sample size:
   a) Justified and satisfactory ❋ (■)
   b) Not justified (■)

3) Non-respondents:
   a) Comparability between respondents and non-respondents characteristics is established, and the response rate is satisfactory ❋ (■)
   b) The response rate is unsatisfactory, or the comparability between respondents and non-respondents is unsatisfactory (■)
   c) No description of the response rate or the characteristics of the responders and the non-responders (■)

4) Ascertainment of exposure:
   a) secure record (e.g. surgical records) ❋ (■)
   b) structured interview (■)
   c) written self report (■)
   d) no description (■)

Comparability

1) Comparability of subjects in different outcome groups on the basis of the design or analysis. Confounding factors are controlled.
   a) study controls for age and sex ❋ (■)
   b) study controls for any additional factor ❋ (■)
   c) No description (■)

Outcome

1) Assessment of outcome:
   a) independent blind assessment ❋ (■)
   b) record linkage ❋ (■)
   c) self report (■)
   d) no description (■)

2) Statistical test:
   a) The statistical test used to analyse the data is clearly described and appropriate, and measurement of the association is presented, including confidence intervals and the probability level (p value) ❋ (■)
   b) The statistical test is not appropriate, not described or incomplete (■)

Figure S1. T_{peak}-T_{end} interval is longer in patients who died compared to survivors in prospective cohort studies [31,32,34,38,41–43,46,50–52].

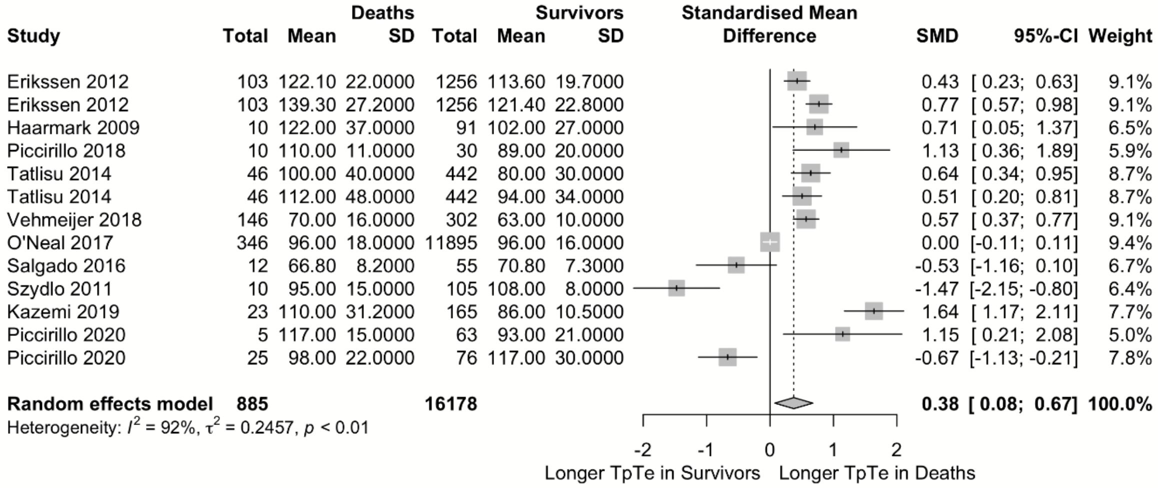

Figure S2. T_{peak}-T_{end} interval is longer in patients who died compared to survivors in studies with a Newcastle-Ottawa-Scale greater than six [27,30,31,32,35,38,39,41–43].

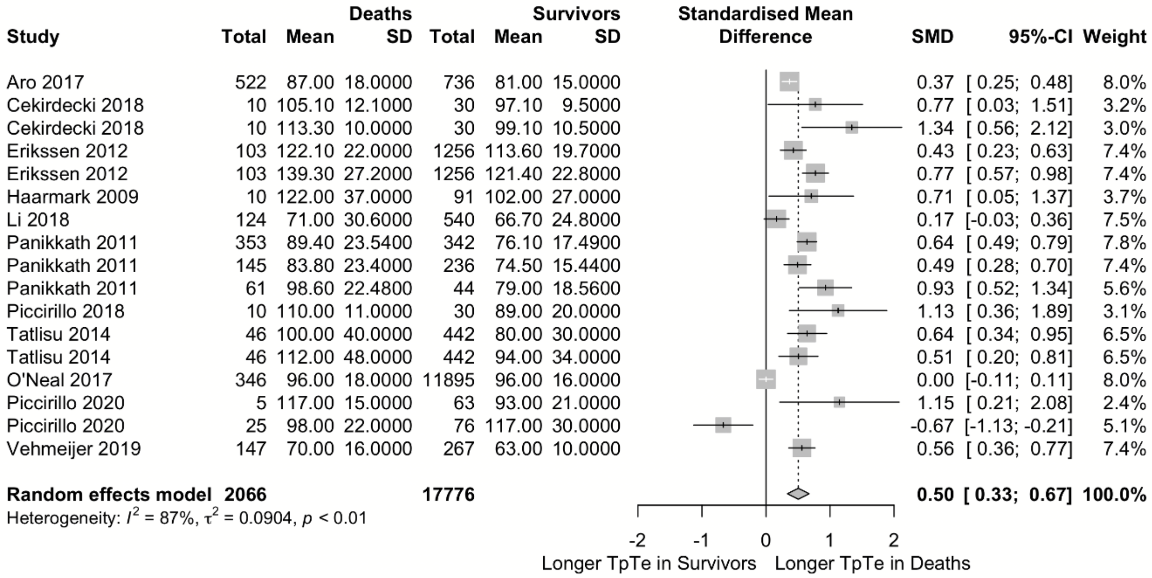

Figure S3. A long T_{peak}-T_{end} interval is associated with all-cause in-hospital death in prospectice cohort studies only [26,48,51,54].

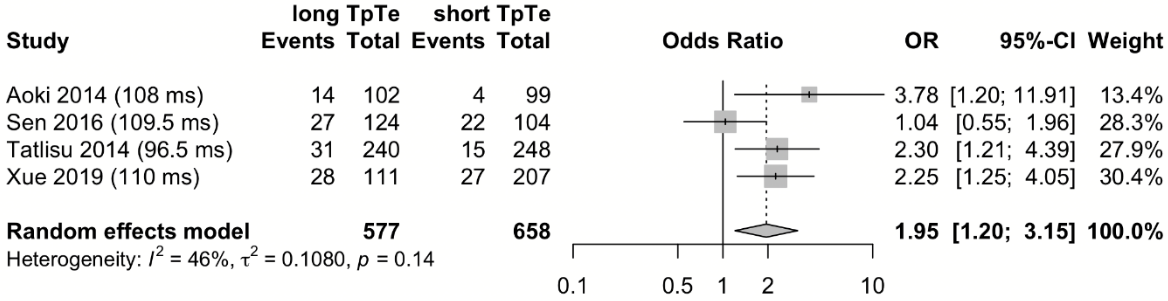

Figure S4. A long T_{peak}-T_{end} interval is associated with all-cause in-hospital death in studies with an Newcastle-Ottawa-Scale greater than 6 [27,33,39,47,48,51].

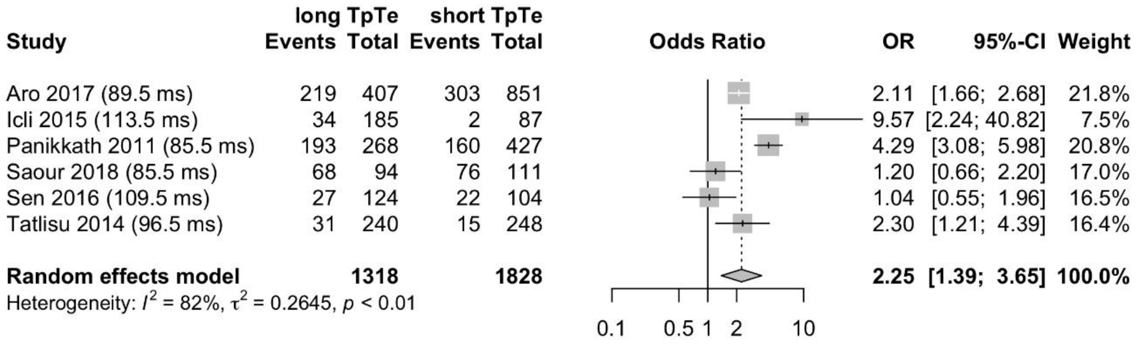

Supplement: Supplementary file 1 [file jcm-12-00992-s001.zip › jcm-2125554-supplementary.pdf]
